# The Third Hand of Neurosurgeons – a novel intraoperative malleable adjustable continuous suction tube

**DOI:** 10.3389/fneur.2023.1333665

**Published:** 2024-01-11

**Authors:** Jibo Zhang, Pan Nie, Zixuan Wei, Chengshi Xu, Xiang Li, Jincao Chen

**Affiliations:** ^1^Department of Neurosurgery, Zhongnan Hospital of Wuhan University, Wuhan, China; ^2^Brain Research Center, Zhongnan Hospital of Wuhan University, Wuhan, China

**Keywords:** microneurosurgery, continuous suction tube, clear surgical fields, The Third Hand of Neurosurgeons, reduce intracranial pressure

## Abstract

**Objective:**

We designed a novel intraoperative malleable adjustable continuous suction tube to obtain clear surgical fields, reduce intracranial pressure, and lower the temperature of the surgical area.

**Methods:**

This device consists of six parts: continuous suction tube head and cotton patty, suction tube, fixed wire position, fixed clip, spiral plastic pressure regulating valve, and tail. It can continuously extract blood, cerebrospinal fluid, and rinsing solution from surgical fields, with minimal contact and trauma to tissues, nerves, and blood vessels, while also having a negligible impact on the surgeon’s focus and procedure.

**Result:**

The excellent and safe performance (simple, malleable, adjustable, space-saving, inexpensive, safe, and effective) of this device in clearing the operating field has been proven in more than 2000 neurosurgical operative procedures. We encountered no complications associated with this device, such as cerebral hematoma, postoperative low intracranial pressure, or vascular and nerve injuries.

**Conclusion:**

The newly innovated intraoperative malleable adjustable continuous suction tube is effective and safe for microneurosurgery.

## Introduction

In microneurosurgery, a clear surgical field, relatively reduced intracranial pressure, and lowered temperature resulting from electrocoagulation hemostasis in the surgical area are imperative for successful and safe neurosurgical procedures, particularly for surgeries that require the protection of some nerves and blood vessels (1–6). We are aware of the challenges posed by the unique anatomical characteristics of brain tissue. The delicate nature of brain tissue, nerves, and blood vessels makes them more susceptible to damage. Additionally, the brain tissue is immersed in cerebrospinal fluid, and the rich blood supply to the brain contributes to an increased presence of fluid in the surgical field. Especially in certain surgeries with very small operating space, the role of assistants will be weakened, so an intraoperative malleable adjustable continuous suction tube is particularly needed.

In order to meet the increasing demand for effective solutions, various methods, such as suction and/or irrigation systems, have been reported in the past few decades (2, 3, 5, 7). However, most of them are complex or infeasible and have not been promoted. We believe that this optimal method can continuously extract blood, cerebrospinal fluid, and rinsing solution from surgical fields, with minimal contact and trauma to tissues, nerves, and blood vessels, while also having a negligible impact on the surgeon’s focus and procedure. To this end, we innovated a novel intraoperative malleable adjustable continuous suction tube. We call it The Third Hand of Neurosurgeons. It has been successfully applied in neurosurgical operative procedures.

## Materials and methods

**Figure 1:**
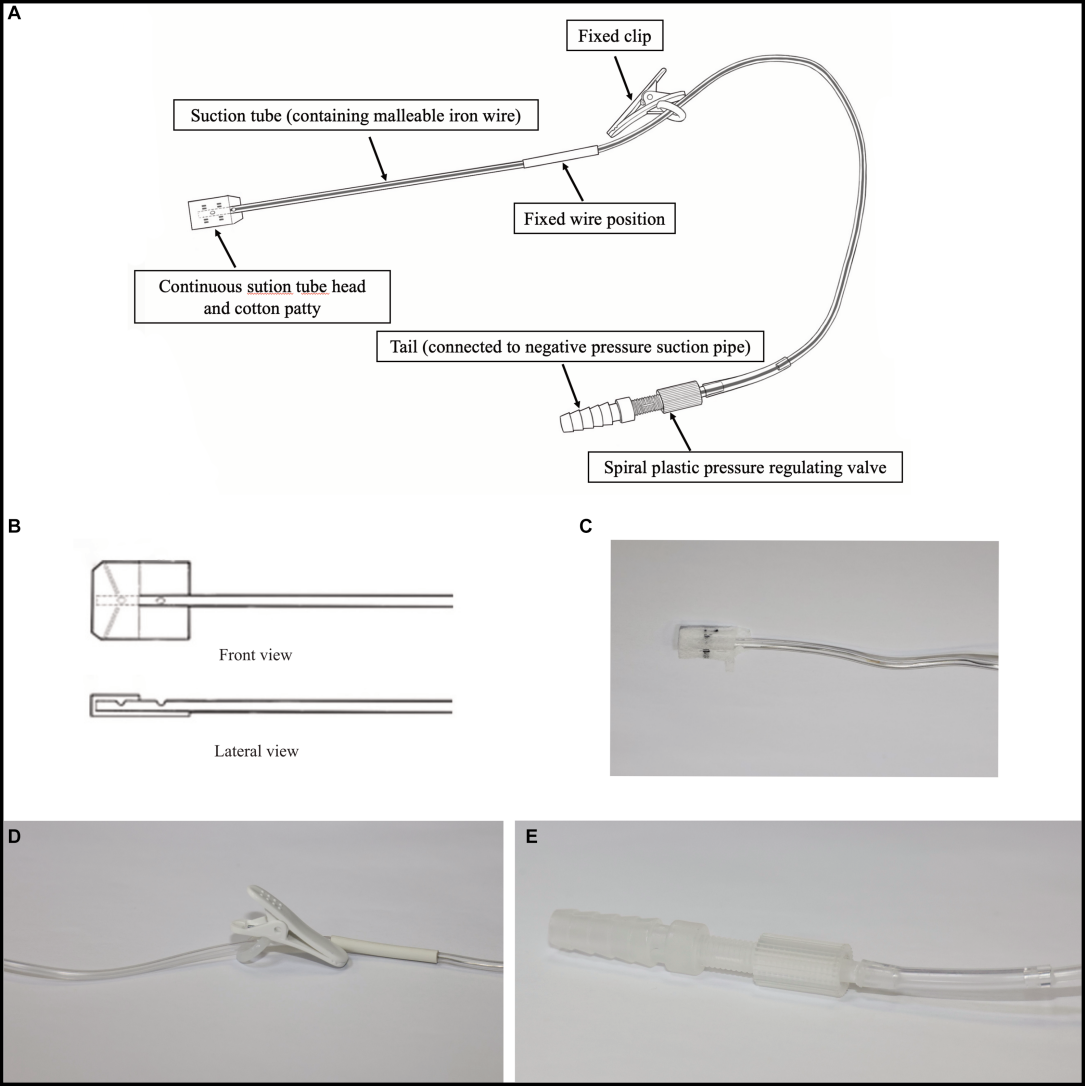
The principal parts of this device. **(A)** This device consists of six parts; **(B)** and **(C)** Continuous suction tube head and cotton patty; **(D)** Fixed wire position and fixed clip; **(E)** Spiral plastic pressure regulating valve and tail.

**Figure 2:**
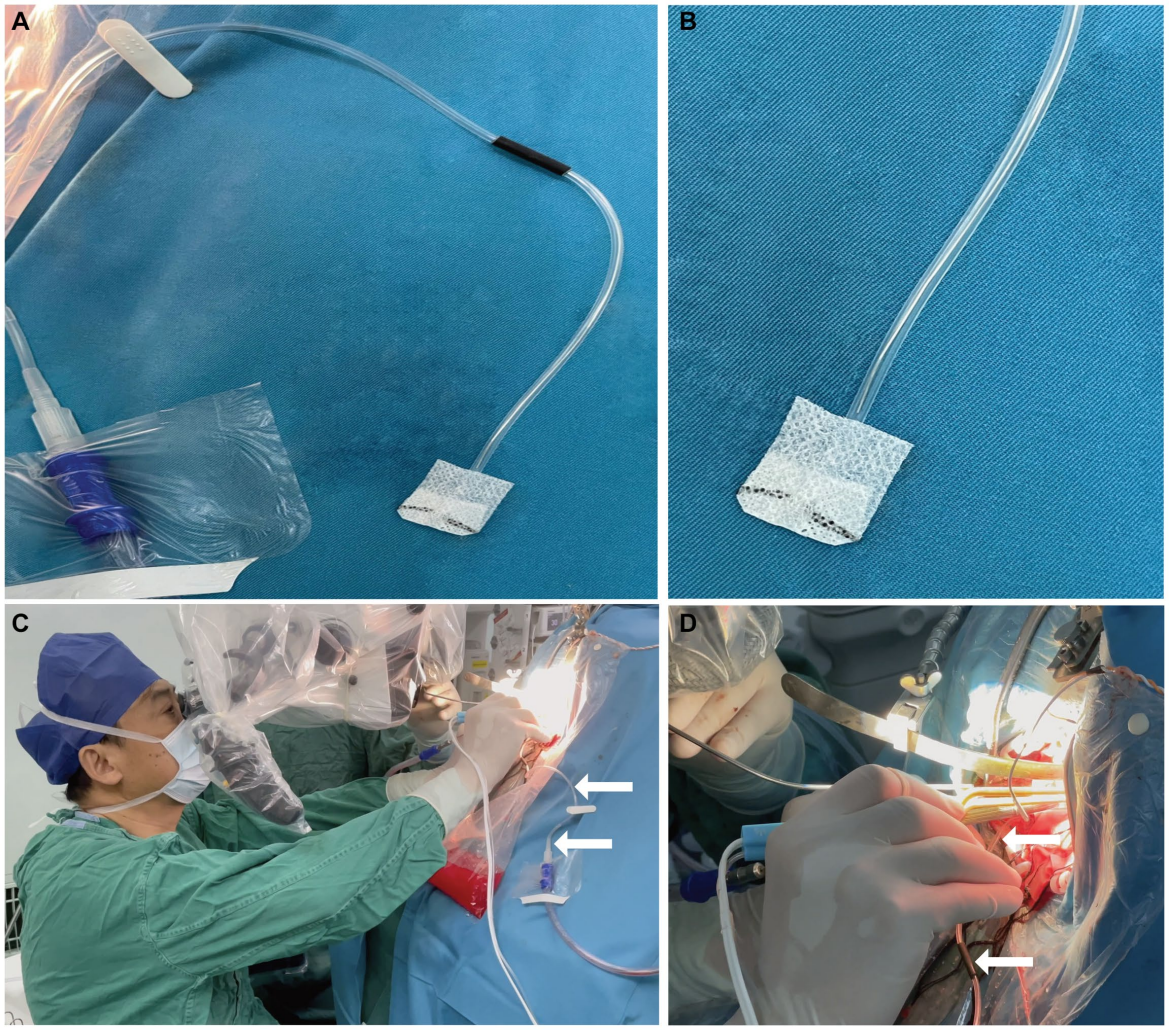
Before and during use of this device. **(A)** After the neurosurgeon disinfects and spreads sterile surgical towels, the neurosurgeon connects the negative pressure suction tube and then connects the tail of the malleable adjustable continuous suction tube to it. The neurosurgeon fixes the malleable adjustable continuous suction tube next to the surgical field; **(B)** Continuous suction tube head and cotton patty; **(C)** and **(D)** When it is needed during surgery, the neurosurgeon needs to adjust the fixed position, then adjust the required pressure, and place the head of the malleable adjustable continuous suction tube into the surgical field.

Figure 1 illustrates the key components of this device. It consists of six parts (Figure 1A): continuous suction tube head and cotton patty (Figures 1B,C, 2B), suction tube (containing malleable iron wire), fixed wire position (Figure 1D), fixed clip (Figure 1D), spiral plastic pressure regulating valve (the pressure can be adjusted by adjusting the size of the air inlet) (Figure 1E), and tail (connected to negative pressure suction pipe) (Figure 1E). The suction tube is made of plastic, characterized by a soft texture, with a diameter of approximately 2 mm. It contains malleable iron wire internally, allowing for convenient manipulation and achieving various degrees of plastic bending.

After disinfecting and laying out sterile surgical towels, the neurosurgeon connects the tail end of the adjustable continuous suction tube to the standard negative pressure suction tube in the operating room and fixes the malleable adjustable continuous suction tube next to the surgical field (Figure 2A). When it is needed during surgery, the neurosurgeon needs to adjust the fixed position, then adjust the required pressure, and place the head of the malleable adjustable continuous suction tube into the surgical field (Figures 2C,D).

**Figure 3:**
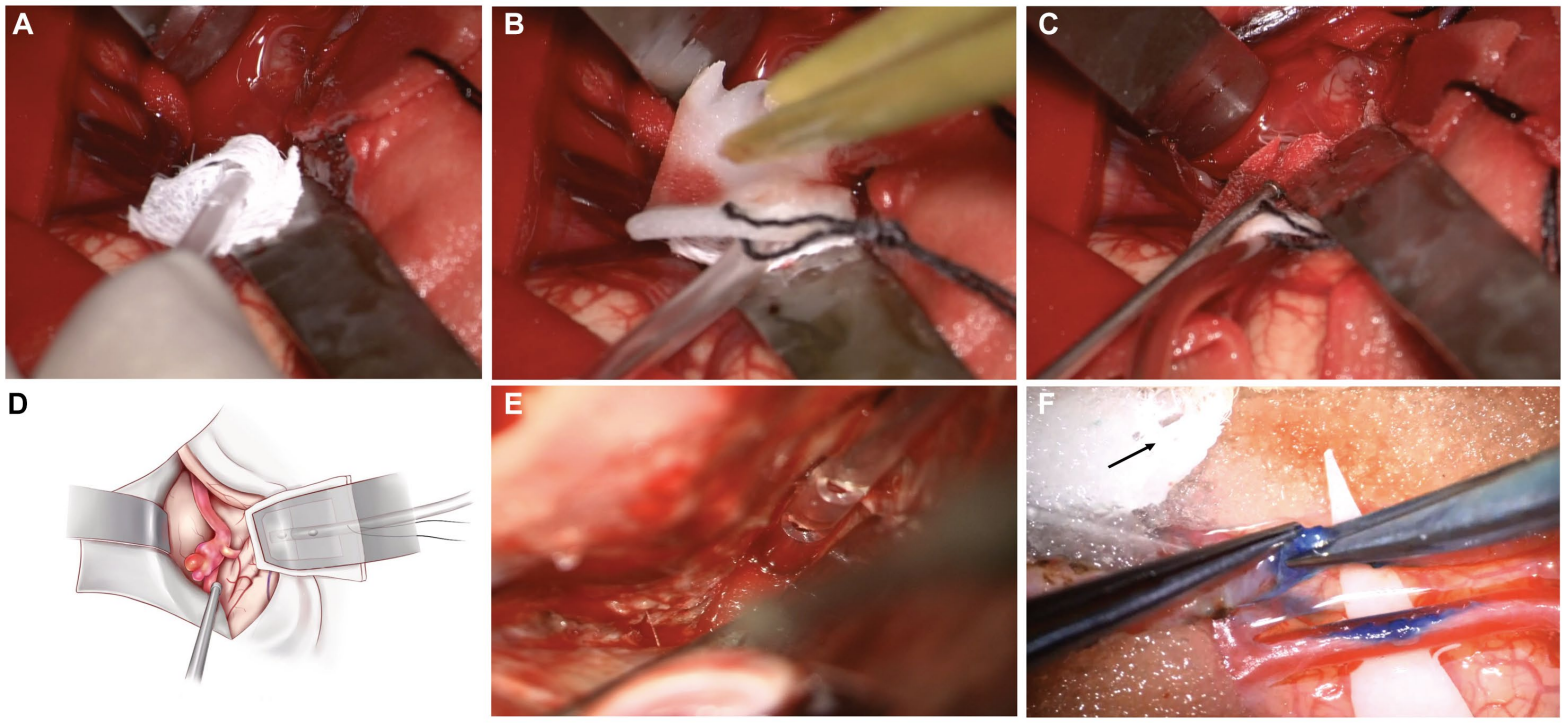
The specific sequence in the surgical field of this device and intraoperative usage **(A)** The specific sequence in the surgical field is to first place the head of the malleable adjustable continuous suction tube in the desired position; **(B)** Then place the brain pad on it; **(C)** Then try fixing it with a brain pressure plate (optional operation). **(D)** Intraoperative usage of this device (Concept Map); **(E)** During surgery, when using an electric drill, to prevent the continuous suction tube head cotton patty from getting entangled in the drill, the cotton patty can be removed and only plastic heads can be used; **(F)** Intraoperative usage of this device (STA-MCA bypass for Moyamoya disease).

**Figure 4:**
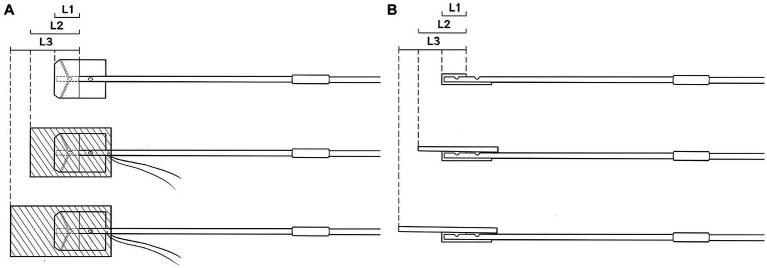
Covering the suction tube with brain sponges of varying sizes helps minimize the frequency of repositioning the suction tube during the surgery. **(A)** Frontal View; **(B)** Lateral View.

The specific sequence in the surgical field is to first place the head of the malleable adjustable continuous suction tube in the desired position (Figure 3A), then place the brain sponges on it (Figure 3B), and then try to fix it with brain pressure plate (optional operation) (Figure 3C). In surgery, we can use brain sponges of varying sizes to reduce the frequency of moving the suction tube, as shown in Figure 4. Intraoperative usage of this device is shown in Figures 3D,F. During surgery, when using an electric drill, to prevent continuous suction tube head cotton patty from getting entangled in the drill, the cotton patty can be removed and only plastic heads can be used (Figure 3E). The device is designed for single-use only. If there is a blockage or a decrease in drainage effectiveness during the procedure, a new one should be replaced.

## Results

The innovative intraoperative malleable adjustable continuous suction tube has been successfully applied in over 2000 neurosurgical procedures, such as brain and spinal tumor resection, aneurysms clipping, extracranial-intracranial bypass for Moyamoya disease, and extirpation of arteriovenous malformations. The device demonstrates excellent and safe performance and is simple, malleable, adjustable, space-saving, inexpensive, and effective. It can continuously extract blood, cerebrospinal fluid, and rinsing solution from surgical fields to achieve clear surgical fields, reduce intracranial pressure, and lower the surgical area’s temperature resulting from electrocoagulation hemostasis. This is accomplished with minimal contact and trauma to tissues, nerves, and blood vessels, ensuring a negligible impact on the surgeon’s focus and procedure. Simultaneously, this device can also streamline surgical procedures, minimize intraoperative bleeding, hasten postoperative patient recovery, shorten hospital stays, and, consequently, decrease overall hospitalization costs for patients.

## Discussion

We innovated a novel intraoperative malleable adjustable continuous suction tube that is simple, malleable, adjustable, space-saving, inexpensive, safe, and effective. We affectionately refer to this device as “The Third Hand of Neurosurgeons.” It addresses a crucial demand in microsurgery and is anticipated to be valuable and intriguing for surgeons practicing in the field.

We all know an intraoperative malleable adjustable continuous suction tube is particularly needed in order to receive a clear surgical field of vision in neurosurgical operations. Several methods, such as suction and/or irrigation systems, have been reported in the past few decades (2, 3, 5, 7). Certain suction-irrigation systems are tailored for specific purposes, such as swiftly clearing the operative field through the control of irrigation fluid volume and suction power or via dual irrigation-suction systems. Nevertheless, the most efficient approach might involve the continuous suction of intraoperative bloody fluids.

Ryu et al. (7) reported a continuous suction system which is a malleable tube made of ultrathin stainless-steel foil sandwiched between ultrafine stainless-steel wire mesh woven tubes. It has been successfully used in neurosurgical operative procedures. Compared to this device, our design has more advantages: it is simpler and cheaper (mainly composed of plastic pipes and iron wires); it is safer (it is disposable, non-reusable, and has a head with cotton patty, which can prevent direct damage to brain tissue, nerves, and blood vessels in the head); it is more effective (has a spiral plastic pressure regulating the valve and it adjusts pressure based on the fluid volume in the surgical field and determines the rate of fluid clearance. It has a transparent plastic tube, that turns red after inhaling bloody liquid, which is consistent with the surrounding tissue color, thereby having a negligible impact on the surgeon’s attention); it is more substantial (in addition to the iron wire that can be shaped and fixed, there is also a fixed clip to further ensure that the device will not be accidentally moved during surgery).

Although we have always pursued a clear surgical field, it does not mean completely dry. Because a completely dry surgical field can be harmful to the brain tissue, nerves, and blood vessels, it is necessary to maintain their moisture through discontinuous irrigation. Therefore, during surgery, an assistant is needed to assist with irrigation and adjusting the pressure of continuous suction by adjusting the size of the spiral plastic pressure regulating valve inlet in order to provide a semi-wet and bloodless operative field.

These characteristics denote that this novel intraoperative malleable adjustable continuous suction tube is completely safe. It has been applied in more than 2000 neurosurgical operations and has demonstrated excellent and safe performance, as well as being simple, malleable, adjustable, space-saving, inexpensive, and effective. It can continuously extract blood, cerebrospinal fluid, and rinsing solution from surgical fields to achieve clear surgical fields, reduce intracranial pressure, and lower the surgical area’s temperature resulting from electrocoagulation hemostasis. From our experience, continuous suction using this device has proven effective for various types of microneurosurgery (see Supplementary Video S1).

## Conclusion

We invented a novel intraoperative malleable adjustable continuous suction tube that is simple, malleable, adjustable, space-saving, inexpensive, safe, and effective for microneurosurgery. We affectionately refer to this device as “The Third Hand of Neurosurgeons.”

## Data availability statement

The raw data supporting the conclusions of this article will be made available by the authors, without undue reservation.

## Ethics statement

The studies involving humans were approved by hongnan Hospital of Wuhan University. The studies were conducted in accordance with the local legislation and institutional requirements. The participants provided their written informed consent to participate in this study.

## Author contributions

JZ: Conceptualization, Formal analysis, Funding acquisition, Project administration, Writing – original draft. PN: Formal analysis, Investigation, Software, Writing – original draft. ZW: Data curation, Methodology, Writing – review & editing. CX: Formal analysis, Writing – review & editing. XL: Conceptualization, Resources, Writing – review & editing. JC: Conceptualization, Methodology, Project administration, Supervision, Writing – review & editing.
